# In-vitro evaluation of Polylactic acid (PLA) manufactured by fused deposition modeling

**DOI:** 10.1186/s13036-017-0073-4

**Published:** 2017-09-12

**Authors:** Matthias C. Wurm, Tobias Möst, Bastian Bergauer, Dominik Rietzel, Friedrich Wilhelm Neukam, Sandra C. Cifuentes, Cornelius von Wilmowsky

**Affiliations:** 1 Department of Oral and Maxillofacial Surgery, University Hospital Erlangen, Glueckstrasse 11, 91054 Erlangen, Germany; 2 Institute for Polymer Technology, Friedrich-Alexander-University Erlangen-Nuremberg, Am Weichselgarten 9, 91058 Erlangen, Germany; 3 Departamento de Ciencia e Ingeniería de Materiales e Ingeniería Química, IAAB, Universidad Carlos III de Madrid, Avda. de la Universidad, 30, 28911 Leganés, Madrid Spain

**Keywords:** Fused deposition modeling, Polylactic acid, Osteoblast, Additive manufacturing, 3D printing

## Abstract

**Background:**

With additive manufacturing (AM) individual and biocompatible implants can be generated by using suitable materials. The aim of this study was to investigate the biological effects of polylactic acid (PLA) manufactured by Fused Deposition Modeling (FDM) on osteoblasts in vitro according to European Norm / International Organization for Standardization 10,993–5.

**Method:**

Human osteoblasts (hFOB 1.19) were seeded onto PLA samples produced by FDM and investigated for cell viability by fluorescence staining after 24 h. Cell proliferation was measured after 1, 3, 7 and 10 days by cell-counting and cell morphology was evaluated by scanning electron microscopy. For control, we used titanium samples and polystyrene (PS).

**Results:**

Cell viability showed higher viability on PLA (95,3% ± 2.1%) than in control (91,7% ±2,7%). Cell proliferation was highest in the control group (polystyrene) and higher on PLA samples compared to the titanium samples.

Scanning electron microscopy revealed homogenous covering of sample surface with regularly spread cells on PLA as well as on titanium.

**Conclusion:**

The manufacturing of PLA discs from polylactic acid using FDM was successful. The in vitro investigation with human fetal osteoblasts showed no cytotoxic effects. Furthermore, FDM does not seem to alter biocompatibility of PLA. Nonetheless osteoblasts showed reduced growth on PLA compared to the polystyrene control within the cell experiments. This could be attributed to surface roughness and possible release of residual monomers. Those influences could be investigated in further studies and thus lead to improvement in the additive manufacturing process. In addition, further research focused on the effect of PLA on bone growth should follow.

In summary, PLA processed in Fused Deposition Modelling seems to be an attractive material and method for reconstructive surgery because of their biocompatibility and the possibility to produce individually shaped scaffolds.

## Background

The skeletal reconstruction of continuity defects caused by accidents or oncological resections in the field of maxillofacial surgery is demanding and requires the use of modern surgical techniques with respect to the size of the defect. The transplantation of autologous tissue has been established as standard in these cases [1]. The resulting problems are a limitation of the transplanting tissue and donor site morbidity. Symptoms range from chronic pain and numbness to restricted movability of the affected regions. The aim of the reconstruction is a restoration of the patients´ appearance with the minimal possible impact on the patients´ chewing functionality and speech.

There is a need for synthetic graft materials which offer good mechanical properties and interfacial biocompatibility. Polylactic acid (PLA) is a promising thermoplastic polymer to be used as a new material in additive manufacturing. Nowadays it is used for osteosynthesis and its characteristics have been considered as an ideal biomaterial for load bearing applications [2]. PLA is well investigated and has been proven to be safe in clinical applications [3]. Due to the fact that patients present individual and complex defects, the material needs to match those needs. Therefore, even complex shapes should be easily fabricated. PLA seems to be a material fulfilling those requirements and has consequently caught a lot of attention in medical technology [3–5]. Besides well controllable degradation timescales and a manufacturing process that allows almost any imaginable shape, PLA offers excellent biocompatibility [5].

Medical devices are typically produced by conventional manufacturing methods like injection moulding. Hence a moulding form needs to be fabricated first. To meet the demands of individually shaped implants for reconstructive surgery, a more flexible manufacturing technique is needed. With earlier production techniques like solvent casting or melt moulding defined pore structures could be obtained [6]. Nonetheless they lack any long-range channelling microarchitecture [6–8].

With respect to direct production of individually shaped implants, additive manufacturing technologies such as FDM can be seen as an ideal production technology [9]. FDM has caught a lot of attention in recent media as 3D printers are getting more popular. FDM is a widely used additive manufacturing technology that uses any thermoplastic (ideally amorphous) material in filament form to build 3d objects layer-by-layer (additive) [10]. Therefore, it opens a wide range of applications in the engineering field. Many attempts have been done to extend this technique in clinical and medical applications for the development of medical implants and scaffolds [11]. Petropolis et al. showed that FDM created models offer sufficient dimensional accuracy for use in maxillofacial surgery [12]. As FDM has several material requirements Guo et al. used templated FDM to produce scaffolds with an almost 100% interconnectivity [13]. Thus the scaffolds lack irregular pores of trabecular bone.

FDM technology is viable for the fabrication of complex mandibular models used for reconstructive surgery and first results are promising [14]. The technique has been used in maxillofacial and mandibular surgical planning and prosthesis design. It has provided virtual operation models to plan the surgery and to optimize the design of the implants before a surgical intervention. FDM has also demonstrated to be an appropriate technique in the fabrication of scaffolds - using any biomaterial as long it is available in filament form and fulfills the process requirements - for medical applications [15–17]. Furthermore, FDM machines offer various configuration options to influence miscellaneous material properties. The combination of a clinically well proven polymer and a flexible manufacturing technique seems promising for its use in reconstructive surgery.

In recent papers of Rietzel et al., they have shown that the interaction between manufacturing process (e.g. nozzle temperature and pathway generation) and material in the FDM process influences the part properties (e.g. crystallinity and thus thermo-mechanical properties) of generated PLA samples [18]. In a study from Patricio et al. a biomanufacturing system called BioCell Printing was used to compare scaffolds produced with solvent casting or melt blending. They showed that PCL/PLA scaffolds produced with solvent casting offered better properties for living cells [19]. It is well known that the final properties of a material do not only depend on the material itself but also on its processing conditions. In order to obtain an adequate melt viscosity of PLA during FDM fabrication process, relatively high temperatures are needed and also the material experiences high shear rate and stress while passing through the nozzle. These challenging conditions during FDM processing could compromise the material biocompatibility as they could induce PLA degradation.

This study aimed to clarify if processing PLA by fused deposition modelling has an influence on its well-known biocompatibility. Due to the increased availability of various new PLA types the results of this paper are a fundamental basis for further investigations in that field.

## Methods

### Creating PLA scaffolds

**Fig. 1:**
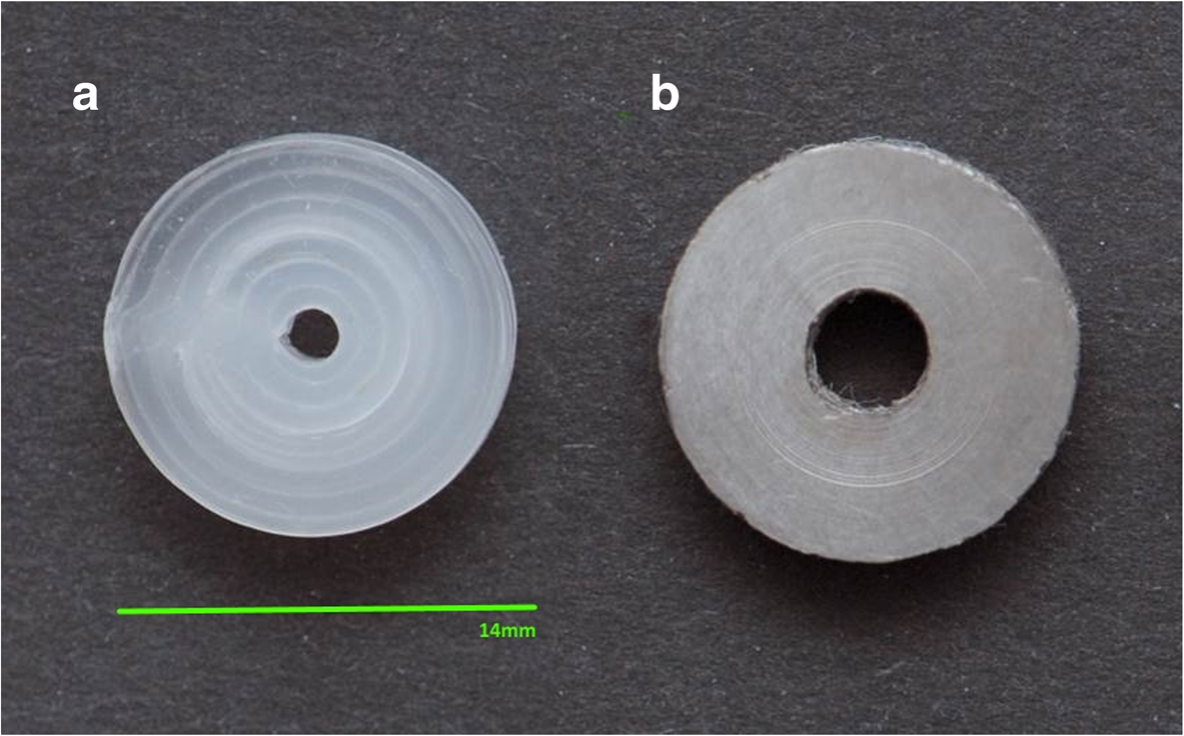
PLA-Sample (**a**) and Titan control (**b**). Diameter approximately 14 mm, height 4 mm

For our study, we used Polylactide Biomer® L9000 (Biomer, Germany). This material is a semi-crystalline biopolymer with a glass transition temperature around 55 °C ± 2 °C and a melting point around 165 °C ± 0.5 °C, its melt flow index is within the range 3.0–6.0 g/10 min. Its properties allow PLA to be processed in a stable way to thin filaments and fulfills the FDM process requirements. According to the manufacturers´ requirements Polylactide Biomer was first dried and then extruded to filaments (diameter = 1.65 mm ± 0.05 mm) in a micro extruder (ED-N20-25D, Extrudex Kunststoffmaschinen GmbH). The obtained filaments were processed in a Stratasys FDM 8000 machine with a nozzle temperature of 225 °C to three-dimensional discs with a diameter of approximately 14 mm, a height of 4 mm and a cylindrical hole in the center of approximately 2 mm (Fig. 1). The crystallinity of the FDM processed part was determined by measuring the heat of fusion and heat of recrystallization from differential scanning calorimetry tests according with the next equations:

$$ \varDelta {H}_{total}=\varDelta {H}_{melt}-\varDelta {H}_{recrystallization} $$

$$ {f}_c=\frac{\varDelta {H}_{total}}{\varDelta {H}_m^0}\times 100 $$

where $\varDelta {H}_m^0$ is the melting enthalpie of a fully crystalline PLA (93.0 J/g) [20]. The PLA samples manufactured by FDM presented a crystalline fraction of 22% ± 0.04%.

### Titanium discs

Titanium disks (5 mm thick, 11 mm diameter) were prepared by electron beam melting (EBM) of a commercially available Ti-6Al-4 V powder (particle size 45–100 lm) using EBMS12 system (Arcam AB, Mo¨lndal, Sweden). The process is described in detail by Heinl et al. [21].

### Cell culture

For our research, we used human fetal osteoblasts (hFOB 1.19). The cells were cultured in 175 cm2 flasks (Greiner bio-one, Germany) with DMEM-F12-medium (Invitrogen, Germany) supplemented with 10% foetal calf serum (PAA Laboratories, Germany), 105 IU penicillin and 100 mg/L streptomycin (Invitrogen, Germany) at 34 °C and 5% CO2. At a confluence of 80% the cells were harvested, washed with phosphate-buffered saline (PBS), counted and 1 × 10^4^ cells were seeded onto every specimen.

### Cell viability

As polystyrene is the common flask material, cell viability was only compared between titanium and PLA discs. With a combined staining of fluorescein diacetate (FDA) (10 μg/mL in PBS. Invitrogen, Germany) and propidium iodide (PI) (50 μg/mL in PBS, Invitrogen, Germany) we investigated cell viability. Twenty-four hours after cell seeding culture medium was removed and samples were covered with FDA/PI dye for 20 min. After carefully washing with PBS, samples were observed with an inverse microscope (Axioskop, Zeiss, Germany). Cell viability was quantified by counting number of living and dead cells for each sample at three different regions of interest with a 10×/0.3 objective (Plan-Neofluar, Zeiss, Germany).

### Cell morphology

Cell morphology on PLA samples and titanium control was examined using scanning electron microscopy. The samples were carefully washed with PBS, then fixated in fixating solution 1 (5 ml glutaraldehyde, 20 ml paraformaldehyde, 0.3 g sucrose) at 4 °C for 2 h. Afterwards rinsed three times with washing buffer (1:1 Deionized water and Sorensen’s phosphate) and then fixated with fixating solution 2 (1:1 4% Osmiumsolution and Sorensens’s phosphate) at 4 °C for 90 min. The samples were then washed with deionized water and then dehydrated with increasing concentrations of acetone (30, 40, 50, 60, 70, 90, 95 and 100%) for 10 min each. 100% acetone was changed twice. Further the cells were dried with hexamethyldisilazane (Sigma, Germany). SEM imaging (XL30 Scanning Electron Microscope, Phillips, Eindhoven, The Netherlands) was conducted at voltages ranging from 5 to 30 kV after the samples surfaces were gold sputtered.

### Cell proliferation

For cell proliferation we compared PLA, polystyrene and titanium. We determined cell proliferation by determining the number of living cells after 1, 3, 7 and 10 days. At given time points cells were detached with Trypsin (Invitrogen, Germany), washed with PBS, resuspended and counted with Casytron cell counter (Schärfe Systems, Germany).

### Statistical analysis

All measurements were performed at least five times and expressed as mean and standard deviation. For the analysis, we used SPSS (Version 21.0 for Windows). Analysis of variance (ANOVA) was employed to asses statistical significance of the data. Bonferroni was used for post hoc comparison. Values of *p* < 0,05 were considered to be statically significant.

## Results

### Cell viability

**Fig. 2:**
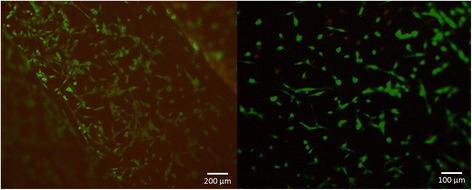
FDA/PI- viability staining of osteoblasts after 24 h PLA (*left*) Titan (*right*). Viable cells are stained *green*, dead cells *red*

**Fig. 3:**
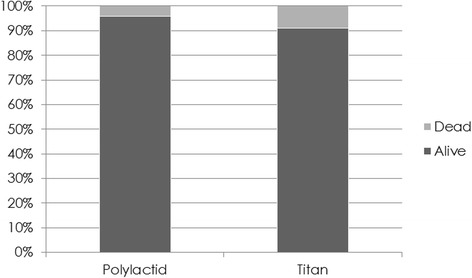
FDI-PI staining performed after 24 h in culture cell. A cell viability of 91.7% ±2.7% for titanium discs and 95.3% ± 2.1% for PLA discs was found

After 24 h in culture cell viability was investigated by FDA/PI staining. A cell viability of 91.7% ±2.7% for titanium discs and 95.3% ± 2.1% for PLA discs was found (Figs. 2 and 3, Table 1).

**Table 1:** Cell viability expressed as mean values and standard deviation. Highest values could be found for osteoblasts growing on Polylactic acid

Samples	Mean values	Standard deviation
Polylactic acid	95.3%	± 2.1%
Titanium	91.7%	± 2.7%

### Cell morphology

**Fig. 4:**
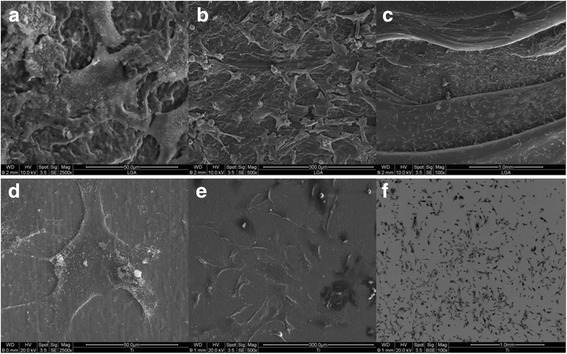
Scanning electron microscopy revealed that PLA disks and Titan control were homogenously covered with regularly spread osteoblasts. Cells were regularly shaped and showed spread filopodia connected to the sample surfaces. PLA samples (**a**) 1 mm (**b**) 300 μm (**c**) 50 μm magnification; Titan control (**d**) 1 mm (**e**) 300 μm (**f**) 50 μm magnification

Scanning electron microscopy revealed that PLA disks and Titanium control were homogenously covered with regularly spread cells. Cells were regularly shaped and showed spread filopodia connected to the sample surfaces (Fig. 4).

### Cell proliferation

**Fig. 5:**
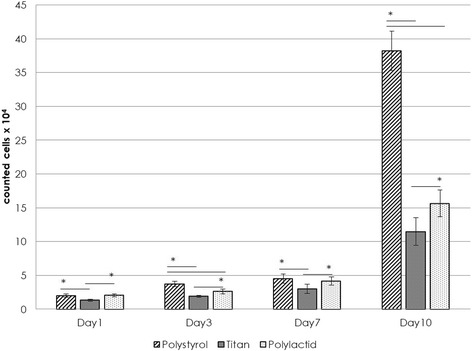
Proliferation of osteoblasts on different materials. The y-axis shows counted cells (× 10^4^) of three different materials at four different times (x-axis). Significant results are marked with a star (*). Values of *p* < 0,05 were considered to be statically significant. Polystyrene showed significant higher cell counts compared to titanium and Polylactid on day 3 and 10. Polylactid showed significant higher proliferation compared to titanium on any day

As shown in Fig. 5, a constant proliferation of osteoblasts could be observed for all samples. On day one 1.8 ± 0.1 × 10^4^ cells were counted on polystyrene control, 1.3 ± 0.1 × 10^4^ cells on titanium control and 2.7 ± 0.5 × 10^4^ cells for PLA samples. Statistical analysis revealed that cell proliferation was significantly higher on Polystyrene compared to titanium (*p* < 0,05) and significantly higher on PLA compared to titanium (*p* < 0,05). On day three the cell number doubled for polystyrene (3.8 ± 0.5 × 10^4^ significant to titanium *p* < 0,05 and significant to PLA *p* < 0,05) and PLA (4.6 ± 5.5 × 10^4^ statistical significance compared to titanium *p* < 0,05), whereas cells on titanium only slightly increased (1.9 ± 0,04 × 10^4^). Day seven showed a strong increase of cell numbers only on polystyrene with 12.392 ± 1.454 × 10^4^ cells. Titanium (2.677 ± 0.9542 × 10^4^) and PLA (5.8 ± 1.1 × 10^4^) remained moderate on day seven. Statistical analysis revealed that cell proliferation was significantly higher on polystyrene compared to titanium (*p* < 0,05) and significantly higher on PLA compared to titanium (*p* < 0,05). Cell proliferation experiments ended after day 10 due to the rapid growth of the cells on polystyrene. Cells growing on polystyrene jumped to 36.3 ± 0.9 × 10^4^ (significant to titanium *p* < 0,05 and significant to PLA *p* < 0,05), cells on titanium to 10.4 ± 3.8 × 10^4^ and cells on PLA samples to 15.5 ± 1.1 × 10^4^ (statistical significance compared to titanium *p* < 0,05). (Table 2, Fig. 5).

**Table 2:** Counted cell numbers on given timepoints. Polystyrene showed highest proliferation rates followed by PLA and titanium

	Polystyrene	Titanium	Polylactic acid
Day 1	1.8 ± 0.1 × 10^4^	1.3 ± 0.1 × 10^4^	2.7 ± 0.5 × 10^4^
Day 3	3.8 ± 0.5 × 10^4^	1.9 ± 0,04 × 10^4^	4.6 ± 5.6 × 10^4^
Day 7	12.4 ± 1.5 × 10^4^	2.7 ± 1,0 × 10^4^	5.8 ± 1.1 × 10^4^
Day 10	36.3 ± 0.9 × 10^4^	10.4 ± 3.8 × 10^4^	15.5 ± 1.1 × 10^4^

## Discussion

Since the amount of autologous tissue for bone reconstruction is highly limited the application of bone substitute materials with matching properties to bone is an area of interest. Especially in the reconstruction of the face the implant individually adapted to the patient’s needs displays a reduction of the psychological strain. Additive Manufacturing permits to produce adapted prostheses inexpensively and individually [8]. Previous materials used in AM such as polyamide 12 are not yet suitable for use in the medical field as implant material. A promising material is PLA – or more specific - its most used two stereoisomers named poly-L-lactide (PLLA) and poly-D-lactide (PDLA) [22]. Whereas pure PLLA has a slow resorption – about 2 years - [23] PDLA loses its mechanical strength faster [24]. Depending on the purpose - e.g. osteosynthesis or bone substitute - PLA offers various opportunities in the medical field. Another advantage is that the E- module of PLA is lower than the E-module of the corticalis. Therefore stress-shielding is reduced [24]. Nonetheless the fitting handling process is not found yet, and it is known that environmental parameters can influence the properties of PLA [25]. Various handling processes have been tried so far but still complete trial series from bench to animal models are rare [14, 18–20, 26, 27]. PLA is considered biocompatible but the effects of FDM in regards to biocompatibility of PLA are unknown so far [3, 14]. Other studies using FDM with polymers but PLA showed no alterations in biocompatibility or osteogenic behavior [28, 29].

The aim of this study was to produce samples of polylactic acid by additive manufacturing and investigate the effect of the prepared samples on human fetal osteoblast in vitro. Nozzle temperature used was 225 °C which led to a crystalline degree of 22%. It is known that the higher the processing temperature the higher the degree of crystallinity which influences the mechanical properties and the resorption behavior of the implant [18]. Knowing this effect and creating a reproducible crystal structure is important for the usability of the material in later research (e.g. in clinical studies). PLA manufactured by FDM with a nozzle temperature of 225 °C presented a modulus of elasticity of 3.2 ± 0.4 GPa in tensile tests. This E-module value falls within the moduli range of trabecular bone in tensile (0.76–10 GPa) and within the lower limit of the moduli range of cortical bone (3.3–20 GPa) [30]. The stiffness of the PLA used should be appropriate for maxillofacial applications.

The studies on cell morphology with SEM exhibited that the cells were spread regularly on the PLA samples as well as on the control samples of titanium and their filopodias were connected to the sample surfaces. This indicates that the process of rapid prototyping does not alter the properties of polylactic acid in a way that would have a cytotoxic effect on cellular growth under the chosen study conditions. A similar result is described in a study by XU et al. [5]. They created PGA/PLA scaffolds and seeded bone marrow stem cells on the scaffolds. Cell adherence was given. Nonetheless the AM method was CAD/CAM (computer-aided design and computer-aided manufacturing) and a mix of PLA and PGA (poly(glycolic acid)) was used, therefore the comparison is misleading but it underlines the biocompatibility. Hsu et al. clarified the possibility of seeding chondrocytes on FDM created PLA –more specific PDLA- scaffolds [31]. They also faced no problems regarding biocompatibility. In a study from Patricio et al. a biomanufacturing system called BioCell Printing was used to compare scaffolds produced with solvent casting or melt blending. They showed that PCL/PLA scaffolds produced with solvent casting offered better properties for living cells [19]. Also the thought behind the use of two polymers seems logic to cope a polymers disadvantage [32], our study aimed to evaluate only one polymer to minimize risk of bias.

The FDA-PI staining (Fig. 3) showed similar high cell viabilities with 95.3% ± 2.1% for the PLA samples and those made of titanium with 91.7% ±2.7%. The cell proliferation on the other hand showed significant differences among the samples. The human foetal osteoblast grew best on polystyrene followed by the PLA samples. The lowest growth was observed on the samples made from titanium. Though it has been shown in earlier studies, that human fetal osteoblasts grow very well on titanium [33] and that polylactic acid and titanium have similar advantages as osteosynthesis material in vivo [34], we found the cell number of osteoblasts growing on PLA samples higher than those growing on titanium. The cell proliferation of osteoblasts growing on polystyrene control samples and PLA samples differed significantly. The difference may possibly be attributed to surface roughness and due to the rough surface osteoblasts do not proliferate as quickly as on polystyrene. Studies have shown that the roughness and the chemical structure of the surface can have an influence on cell proliferation and spreading [33, 35, 36]. But in the findings of these studies are discrepancies regarding the effect of surface roughness on cell proliferation. These inconsistencies may result in the different cell types used, cell culture conditions, different media and fabrication methods [37–39]. Hsu et al. found that the architecture of a PDLA-construct influences the proliferation of chondrocytes [31]. Even though the examined constructs were three-dimensional it could support the thesis that surface properties also influence cell proliferation. Andrukhov et al. described that surface roughness influences cell proliferation, migration and the expression of alkaline phosphatase, osteocalcin and VEGF. No influence was found on the expression of OPG and RANKL [40]. Nonetheless it is only a possible explanation and our aim was not to determine the influence of surface roughness. Another point is that cell culture equipment like polystyrene flasks are usually optimized for in vivo cell proliferation. This may explain the best results of polystyrene within this experiment.

An additional influence on cell proliferation could be the release of residual monomers and non-fused residues from the produced samples into the surrounding medium, which has already been shown for bone cements [41]. This can be accompanied with immune responses to residual monomers and degradation products [26, 42–44], but could not be investigated under chosen study conditions. The results of the cell proliferation and the cell vitality staining suggest that the PLA scaffolds produced by rapid prototyping are biocompatible for osteoblasts. Polylactic acid is an established material for osteosynthesis and due to its characteristics as a thermoplastic polymer it seems suitable for use in the additive manufacturing leading to biocompatible and individually shaped implants.

Titanium was chosen as control because of its good results in previous experiments investigating cellular growth on different titanium surfaces [45]. Although titanium is an established material, titanium suffers another disadvantage. While PLA-based implants allow regular postoperative radiographic controls titanium produces artifacts and therefore restrains radiographic evaluation.

Our aim was to show the biocompatibility of a next generation osteosynthesis and graft material comparable to titanium. The FDA-PI-Staining in combination with the SEM images clearly demonstrates that the rapid prototyped polylactic acid does not induce any cytotoxic effects on osteoblasts and seems to be a candidate for new treatment strategies weather as a carries – e.g. scaffolds – or a osteosynthesis material.

## Conclusions

The manufacturing of PLA discs from polylactic acid using FDM was successful. The in vitro investigation with human fetal osteoblasts showed no cytotoxic effects. Furthermore FDM does not seem to alter biocompatibility of PLA. Nonetheless osteoblasts showed reduced growth on PLA compared to the polystyrene control within the cell experiments. This could be attributed to surface roughness and possible release of residual monomers. Those influences could be investigated in further studies and thus lead to improvement in the additive manufacturing process. In addition further research focused on the effect of PLA on bone growth should follow.

In summary, PLA processed in Fused Deposition Modeling seems to be an attractive material and method for reconstructive surgery because of their biocompatibility and the possibility to produce individually shaped scaffolds.

## Abbreviations

AM: additive manufacturing
EBM: electron beam melting
FDA: fluorescein diacetate
FDM: fused deposition modeling
hFOb: human osteoblasts
OPG: osteoprotegerin
PBS: phosphate-buffered saline
PDLA: poly-D-lactide
PGA: poly(glycolic acid)
PI: propidium iodide
PLA: polylactic acid
PLLA: poly-L-lactide
RANKL: receptor activator of nuclear factor kappa-B ligand
SEM: scanning electron microscope
VEGF: Vascular endothelial growth factor
